# A case of pediatric psoriasis successfully and rapidly treated with ixekizumab

**DOI:** 10.1111/dth.15108

**Published:** 2021-08-31

**Authors:** Matteo Megna, Luigi Fornaro, Mario De Lucia, Orsola Rescigno, Elisa Camela, Gabriella Fabbrocini

**Affiliations:** ^1^ Section of Dermatology ‐ Department of Clinical Medicine and Surgery University of Naples Federico II Napoli

Dear Editor,

Psoriasis is a chronic, inflammatory, and relapsing disease which affects ~1% of children and adolescents and whose onset before 20 years of age occurs in 35%–50% of patients.^1^ Even in childhood, moderate‐to‐severe psoriasis is associated with increased incidence of multiple comorbidities such as psoriatic arthritis, obesity, diabetes, inflammatory bowel diseases, and reduced health related quality of life.^2^ Due to the lack of clinical trials, therapeutic options in pediatric psoriasis are limited. Particularly, conventional systemic treatments are approved only after 18 years of age while phototherapy is time‐consuming showing logistic concerns.^3^ Biologics represent the only officially approved systemic drugs for moderate‐to‐severe pediatric psoriasis. Etanercept (anti‐TNF‐α), ustekinumab (anti IL12/23), secukinumab, and ixekizumab (anti IL17A) have been approved in patients ≥6 years of age while adalimumab (anti TNF‐α) has been approved in patients aged ≥4.^3^, ^4^, ^5^, ^6^ Particularly, in Italy anti‐IL17 and ustekinumab are still waiting reimbursement from the National Health System for pediatric psoriasis. A 17‐years‐old male presented with a 12‐year history of plaque psoriasis also involving the scalp, palms, and genitals [psoriasis area severity index (PASI): 21, body surface area (BSA): 38%, Figure 1A,B]. Clinical lesions were associated with severe pain and discomfort which impaired daily activities and social relationships. Family history of psoriasis was positive. He did not take any drugs nor suffered from other diseases. His failed treatment history was wide: topical agents (0.05% clobetasol propionate), 16 weeks of oral cyclosporine (2.5 mg/kg/day) at the age of 9 years and 24 weeks of nb‐UVB therapy when he was 10 years. Subsequently, the patient started etanercept at the age of 11 years that provided adequate efficacy for 104 weeks when he (at the age of 13 years) was switched to adalimumab for loss of PASI75 response. After an initial optimal response, almost 2 years thereafter, nb‐UVB therapy was added to adalimumab for psoriasis worsening without any significant results for 24 weeks. Similar outcomes were observed for the addition of methotrexate (10 mg/weekly) for 3 months.

**FIGURE 1:**
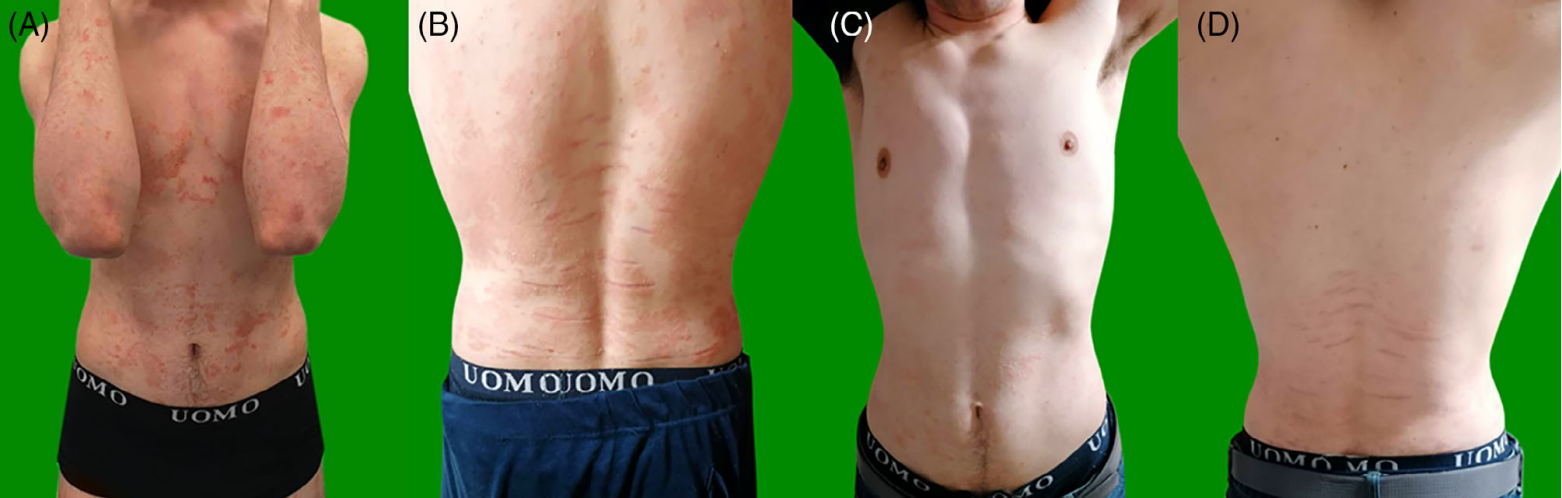
Patient at baseline (A and B) and after 4 weeks of ixekizumab treatment (C and D)

Finally, ixekizumab was started due to disease severity (PASI:21), the huge impact on quality of life, the involvement of difficult to treat areas (scalp, palms, and genital area), and previous failure of all available conventional and biologic systemic treatments for pediatric psoriasis. We observed a complete skin clearance after only 4 weeks of treatment together with a huge improvement in quality of life and skin symptoms (PASI; 0, BSA 0%, Figure 1C,D); these results were maintained up to the last follow up at 24 weeks of treatment. To date in literature, there is only one PHASE III study on ixekizumab for pediatric psoriasis, which demonstrated PASI75 response in 89% at 12 weeks and PASI90 at week 48 in 78%.^4^ Our case seems to confirm ixekizumab rapidity of action in pediatric psoriasis as observed in adults where brodalumab and ixekizumab resulted in the most rapid biologics with PASI90 achieved in 25% of patients after 3.5 and 4.1 weeks, respectively.^6^

To the best of our knowledge, we described the first case of pediatric psoriasis with a complete response in only 4 weeks of ixekizumab. Although limited to a single patient, our experience suggests that ixekizumab might be considered as a possible valid choice for the management of pediatric recalcitrant psoriasis. Further real life studies are required to confirm the efficacy and safety of anti‐IL17 for pediatric psoriasis.

## CONFLICT OF INTEREST

All authors declare no conflict of interest

## AUTHOR CONTRIBUTION

Matteo Megna: have made substantial contributions to conception and design, drafting the manuscript and revising it critically for important intellectual content, and given final approval of the version to be published. Luigi Fornaro: drafting the article, and revising it critically for important intellectual content, and given final approval of the version to be published. Mario De Lucia: bibliography research, acquisition of data, drafting the article. Orsola Rescigno: bibliography research, acquisition of data, photo acquisition. Elisa Camela: drafting the article, and revising it critically for important intellectual content, and given final approval of the version to be published. Gabriella Fabbrocini: have made substantial contributions to conception and design, drafting the manuscript and revising it critically for important intellectual content, and given final approval of the version to be published.

## INFORMED CONSENT

The patient and the mother of the patient (17 years old) gave consent for photo acquisition and publication.

## Data Availability

Data sharing not applicable to this article as no data sets were generated or analysed during the current study.
